# PTH Promotes Chondrogenesis of Fibrocartilage Stem Cells and Alleviates Temporomandibular Joint Osteoarthritis

**DOI:** 10.1007/s13770-025-00723-y

**Published:** 2025-06-16

**Authors:** Zhihang Yue, Wuyi Gong, Haojun Chu, Yongming Li

**Affiliations:** Shanghai Engineering Research Center of Tooth Restoration and Regeneration and Tongji Research Institute of Stomatology and Department of Orthodontics, Shanghai Tongji Stomatological Hospital and Dental School, Tongji University, Shanghai, 200072 China

**Keywords:** Temporomandibular joint osteoarthritis, Fibrocartilage stem cells, Chondrogenic differentiation, Parathyroid hormone, Cathepsin K

## Abstract

**Background:**

Parathyroid hormone (PTH) can promote subchondral bone formation and alleviate temporomandibular joint (TMJ) osteoarthritis (OA), but the effects of PTH on fibrocartilage stem cells (FCSCs) in cartilage surfaces have yet to be studied.

**Methods:**

We established the TMJOA model in rats and administered PTH to treat them. Rat condyles were analyzed using micro-computed tomography, histological, and immunohistochemical staining. To study PTH's effects on FCSCs *in vitro*, we employed quantitative polymerase chain reaction, Western Blot, and immunofluorescence staining. We also constructed the TMJOA model in tdTomato; Cathepsin K (Ctsk)-Cre mice and rescued them with PTH. EdU and immunofluorescence staining were used to measure the proliferation and chondrogenic differentiation of FCSCs *in vivo*. Furthermore, after discectomy, we injected diphtheria toxin (DT) into the Ctsk-Cre; diphtheria toxin receptor (DTR) mice to ablate FCSCs. Afterwards, PTH was injected, and we evaluated the Collagen Type II Alpha 1 (COL2A1)-positive area using immunofluorescence staining.

**Results:**

We successfully developed a TMJOA model, and after treatment with PTH, the rat condyles' BV/TV and Tb. Th increased, and the expression of chondrogenic-related genes was elevated. Additionally, PTH promoted the chondrogenic differentiation of FCSCs *in vitro*. In tdTomato; Ctsk-Cre mice, the Ctsk/EdU and Ctsk/COL2A1 double-positive cells were increased after PTH administration. Moreover, after the ablation of FCSCs by DT, the effects of PTH treatment were notably reduced.

**Conclusion:**

PTH promotes the proliferation and chondrogenic differentiation of condylar FCSCs.

## Introduction

Temporomandibular joint osteoarthritis (TMJOA) is a degenerative disorder of the temporomandibular joint (TMJ) marked by ongoing cartilage breakdown and destruction in the subchondral bone [1]. The restricted capacity for self-repair of condylar cartilage due to the absence of blood arteries, lymphatic vessels, and nerves [2–4] leads to difficulty in reversing the degraded cartilage in TMJOA. Traditional treatments, including conservative and surgical approaches, can slow disease progression but cannot fully restore damaged cartilage and subchondral bone [5]. Recently, endogenous stem cell therapy has gained considerable attention for its potential to repair damaged tissues by recruiting and stimulating endogenous stem cells in situ, making it a promising aspect of clinical research [6]. Fibrocartilage stem cells (FCSCs) have been identified in the surface layers of condylar cartilage and possess the capability to regenerate cartilage and bone [3]. Transplantation of exogenous FCSCs into TMJ-deficient rat models can enhance the cartilage structure [7]. FCSCs represent the optimal source of stem cells for facilitating the *in vivo* repair of TMJ cartilage. Maintaining the FCSC pool has been reported to be crucial for treating TMJOA [8, 9], offering new avenues for cartilage regeneration [10]. However, the challenge remains to regulate the FCSC pool within an inflammatory environment.

Parathyroid hormone (PTH) is a polypeptide comprising 84 amino acids secreted by the parathyroid gland [11]. Previous studies found that PTH can induce the differentiation of osteoblasts and bone formation and alleviate TMJOA [12, 13]. Research also suggested that PTH promoted chondrogenesis of chondrocytes [14], thus alleviating the symptoms of TMJOA. However, most studies have concentrated on its impact on chondrocytes in the cartilage, and the effects of PTH on FCSCs in cartilage surfaces have yet to be studied.

Our study focused on the FCSCs subsets in the surface layer of TMJ cartilage. We established a rat model of TMJOA and demonstrated that local injection of PTH improved cartilage degradation and promoted chondrogenesis *in vivo*. Additionally, we confirmed the presence of PTHR1 on the surface of FCSCs and found that PTH enhanced the chondrogenic differentiation of FCSCs under inflammatory conditions *in vitro*. Furthermore, by using Cathepsin K (Ctsk)-Cre; diphtheria toxin receptor (DTR) mice to selectively deplete FCSCs from the condylar surface, we observed a reduced therapeutic effect of PTH, indicating that the treatment of TMJOA with PTH might be mediated by regulating FCSCs. PTH may be applied as a treatment option for TMJOA by encouraging the proliferation and differentiation of FCSCs.

## Materials and methods

### Animal

Sprague–Dawley (SD) rats were obtained from Beijing SiBeiFu Animal Technology Co. Ltd. Ctsk-Cre, tdTomato, and DTR mice (C57BL/6 background) were obtained from the Shanghai Model Organisms Center, Inc. Only male subjects were selected to eliminate the influence of estrogen. All animals were kept in a specific pathogen-free facility with unrestricted access to food and water, allowing free movement within their enclosures. Any rats or mice that died after surgery were excluded. Six-week-old male SD rats were randomly assigned to Sham + PBS, OA + PBS, and OA + PTH groups. We induced the TMJOA model by partial disc discectomy [15, 16]. The rat TMJ capsules were incised in the OA + PBS and OA + PTH groups, and the lateral one-third of each disc was removed, exposing the condylar surface. In the Sham + PBS group, the joint capsules were exposed while the articular disc remained intact. Four weeks post-surgery, the rats received intra-articular injections of either PTH for the OA + PTH group or an equivalent volume of PBS for the Sham + PBS and OA + PBS groups, continuing for 4 or 6 weeks. The main active component of PTH is a 34-amino-acid sequence located at the amino terminus, which encompasses nearly all its biological functions [11]. So, PTH (1–34) was used in our study to rescue TMJOA. PTH (1–34) was diluted in PBS to 10 nM (40 ng/ml; 40 μl, 0.1 ml/kg), and the rats were administered intra-articular injections every three days [17, 18]. After observation following administration, the rats and mice exhibited good conditions post-PTH injection. Only one mouse died due to hemorrhage and was subsequently excluded from the experiment. All other rats and mice showed no abnormalities.

Eight-week-old male tdTomato; Ctsk-Cre mice were randomly assigned to Sham + PBS, OA + PBS, and OA + PTH groups. Discectomy or sham surgery was performed, similar to the procedures used in rats. Four weeks post-surgery, the Sham + PBS, OA + PBS, and OA + PTH groups received local injections of PBS or PTH, following the same protocol as the rats for an additional four weeks. The mice were administered with EdU 24 h prior to their sacrifice.

Eight-week-old male Ctsk-Cre; DTR mice and DTR mice were administered 100 ng diphtheria toxin (DT) four weeks after partial disc discectomy. Subsequently, the Ctsk-Cre; DTR mice were randomized into OA + PBS and OA + PTH groups, receiving local injections of either PBS or PTH for four weeks. The DTR mice also received PTH injections during this period.

To ensure the consistency and reproducibility of the surgical procedures, all modeling procedures were performed by the same surgeon under a stereomicroscope. Besides, we established a standardized surgical protocol: After anesthetizing the rats and mice with ether, we disinfected and prepared the surgical area. Then, we made an oblique incision at the junction of the outer and middle thirds of the line connecting the external auditory canal and the corner of the eye. The muscle tissue and joint capsule were bluntly dissected to expose the articular disc. We made a T-shaped incision on the disc and excised the lateral one-third of the disc. The excised disc was retained to ensure consistency in the size of the removed disc across all rats. We used antibiotics locally and closed the wound in layers. Postoperatively, we placed the food in easily accessible locations and monitored the animals regularly for recovery. Additionally, we conducted multiple practice sessions and confirmed the success and stability of the modeling through Micro-computed tomography (μCT) analysis.

### μCT analysis

The rat TMJ condyles were fixed in 4% paraformaldehyde (PFA) at 4°C for 2 days, then positioned within a 14 mm diameter scanning tube and imaged via μCT (model μCT50; Scanco Biomedical, Switzerland). After three-dimensional reconstruction, we aligned the condyles to the same orientation based on the position of the internal oblique line to obtain sagittal and top-view images from identical anatomical positions. Representative sagittal images were taken at the middle scan layer in the sagittal plane. Moreover, twenty slices of subchondral bone were reconstructed for statistical analysis, including the bone volume fraction (BV/TV) and trabecular thickness (Tb. Th). The images were selected for presentation in a blinded manner, where the researcher responsible for image analysis was unaware of the groups.

### Histological analysis

The rat TMJ condyles were fixed in 4% PFA at 4 °C for 2 days and decalcified in 10% ethylenediaminetetraacetic acid for 4 weeks. Following dehydration in an automatic dehydrator (Thermo Fisher, USA), they were embedded in paraffin and sectioned to a thickness of 4 μm.

The Safranin O/Fast green staining kit (Solarbio, Beijing, China), Alcian Blue Periodic acid Schiff (AB-PAS) staining kit (Solarbio), and hematoxylin–eosin (HE) staining kit (Beyotime, Shanghai, China) were performed. Three independent experimenters blindly assessed Mankin scores for TMJ cartilage (Table 1). Immunohistochemical staining was performed using an immunohistochemistry staining kit (MXB, Fujian, China) and a DAB kit (MXB). The primary antibodies included Collagen Type II Alpha 1 (COL2A1, 1:200, Boster, Wuhan, China), SRY-box transcription factor 9 (SOX9, 1:200, Proteintech, Wuhan, China), Aggrecan (ACAN, 1:200, Proteintech) and Interleukin-1β (IL-1β, 1:200, Proteintech).

**Table 1:** Mankin Score

Mankin score	
I. Structure	
a. Normal	0
b. Surface irregularities	1
c. Pannus and surface irregularities	2
d. Clefts to transitional zone	3
e. Clefts to radial zone	4
f. Clefts to calcified zone	5
g. Complete disorganization	6
II. Cells	
a. Normal	0
b. Diffuse hypercellularity	1
c. Cloning	2
d. Hypocellularity	3
III. Safranin O staining	
a. Normal	
b. Slight reduction	1
c. Moderate reduction	2
d. Severe reduction	3
e. No dye noted	4
IV. Tidemark integrity	
a. Intact	0
b. Crossed by blood vessels	1

Mouse TMJ samples were gradient dehydrated with 15% sucrose for 24 h and 30% sucrose for 24 h. After embedding in the OCT compound (Sakura, USA), frozen tissue blocks were sectioned into 10 μm cryosections. The Safranin O/Fast green staining kit (Solarbio) was performed. For immunofluorescence staining, the frozen sections were incubated with COL2A1 (1:200, Boster) rabbit anti-mouse primary antibody at 4 °C overnight, followed by a goat anti-rabbit secondary antibody (1:1000, Invitrogen, USA) at 37 °C for one hour. Ultimately, they were stained with DAPI (Beyotime) and mounted with an anti-fade reagent (Invitrogen).

### EdU assay

For the EdU cell proliferation assay, EdU solution (5 mg/mL, 0.06 mg/g, Beyotime) was injected intraperitoneally 24 h before the mice were sacrificed. EdU assay was conducted via the BeyoClick™ EdU Cell Proliferation Kit (Beyotime).

### FCSC isolation and culture

The TMJ condyles of five-week-old rats were separated after death. The superficial layers were dissected and digested using collagenase type I (3 mg/mL; Sigma, USA) and dispase II (4 mg/mL; Roche, Switzerland) at 37 °C for 40 min. After digestion was terminated, the cells were resuspended and seeded into culture dishes. The culture medium contained αMEM (HyClone, USA) augmented with 10% fetal bovine serum (BI, Israel), 1% GlutaMAX (Invitrogen), and 0.1% 2-mercaptoethanol (Gibco).

FCSCs in the TNF-α group and TNF-α + PTH group were treated with recombinant TNF-α protein (peprotech, USA) at 10 ng/ml for 10 h to simulate the inflammatory environment [19]. PTH at 10 nM was added for the first 6 h of 48 h in the TNF-α + PTH and PTH groups [20].

### Multi-lineage differentiation

For osteogenic and adipogenic differentiation, single clones of FCSCs were seeded and cultured using adipogenic or osteogenic induction media (OriCell, Guangzhou, China). After incubation, cells were stained with Oil Red O (OriCell) to assess adipogenesis and Alizarin Red to evaluate osteogenesis.

For chondrogenic differentiation, single clones of FCSCs were cultured in chondrogenic induction media (OriCell) to form pellets. Cryosections were stained with Alcian Blue.

### Cell counting kit-8 (CCK-8) assay

FCSCs at P1 were cultivated in 96-well plates at 2000 cells/well and incubated for 24 h for attachment (5% CO2, 37 °C). 0/0.1/1/10/20/50 nM PTH were added for six hours. Then, 10 μl of CCK-8 reagent (Yeasen, China) was added to each well. After 4 h of incubation, the OD values were measured at 450 nm to calculate cell viability. The cell viability was calculated using the following equation: Cell viability (%) = (OD treated/OD control) × 100.

### Real-time quantitative polymerase chain reaction (qPCR)

Total RNA was isolated utilizing the RNAiso Plus reagent (TaKara Bio, Japan). Reverse transcription was performed utilizing the PrimeScript™ RT Reagent Kit (TaKara Bio). qPCR was analyzed using a LightCycler System (Roche). Fold changes in mRNA expression were quantified using the 2^−ΔΔCt^ technique and normalized relative to GAPDH expression. The sequences of primers are enumerated in Table 2.

**Table 2:** Genes and primer sequence

Gene	5′-3′ sequences
GAPDH	F AGGTCGGTGTGAACGGATTTG
	R GGGGTCGTTGATGGCAACA
COL2A1	F TGTATGGAAGCCCTCGTCCT
	R TGCCCCTTTGGCCCTAATTT
ACAN	F TTCCACCAGTGCGATGCAG
	R TGGTGTCCCGGATTCCGTA
PRG4	F CTGTGGAGAGGGTTCCCAAA
	R CAGGTATAAGCCATGCAATGGGA
IL-1β	F GCAACTGTTCCTGAACTCAACT
	R ATCTTTTGGGGTCCGTCAACT
MMP13	F CTTCTTCTTGTTGAGCTGGACTC
	R CTGTGGAGGTCACTGTAGACT
TNF-α	F AGCCGATGGGTTGTACCTTG
	R ATAGCAAATCGGCTGACGGT

### Western blot

FCSCs were lysed in RIPA buffer (Beyotime) augmented with protease inhibitors, phosphatase inhibitors, and EDTA in a ratio of 100:2:2:2. The samples were blended with 5 × loading buffer (Sangon, Shanghai, China) and heated at 100 °C for 5 min. Then, proteins were isolated using SDS-PAGE electrophoresis and transferred to 0.22 μm PVDF membranes (Millipore, USA). The membranes were incubated with primary antibodies at 4 °C overnight. Then, they were rinsed with TBST and incubated with secondary antibodies for an hour. Blots were visualized by ImageQuant 800 (Cytiva, USA). The primary antibodies included COL1A1 (1:2000, Boster), COL2A1 (1:2000, Boster), SOX9 (1:2000, Proteintech), Proteoglycan 4 (PRG4, 1:1000, Abclonal, China), and GAPDH (1:3000, Affinity, USA).

### Flowcytometry analysis

FCSCs were incubated for 30 min with primary antibodies: CD90-FITC, CD29-Pacific Blue, CD11b/c-PE, and CD45-Percp-cy5.5 (1:200, Biolegend, USA). Flow cytometry experiments were conducted using the BD LSR Fortessa (BD, USA), and the results were analyzed with FlowJo v10.8.1.

### Statistical analysis

The Kolmogorov–Smirnov test was employed for the normality test. Student t-test or Mann–Whitney U-test was used based on the normality of the two sets of continuous variables. For comparisons involving multiple sets of continuous variables, either a one-way analysis of variance or the Kruskal–Wallis test was utilized. Statistical analyses were conducted utilizing SPSS (v22.0) and GraphPad Prism 8 software. All data are expressed as mean ± standard deviation with a *p* value < 0.05 being statistically significant.

## Results

### Partial discectomy induces TMJOA

**Fig. 1:**
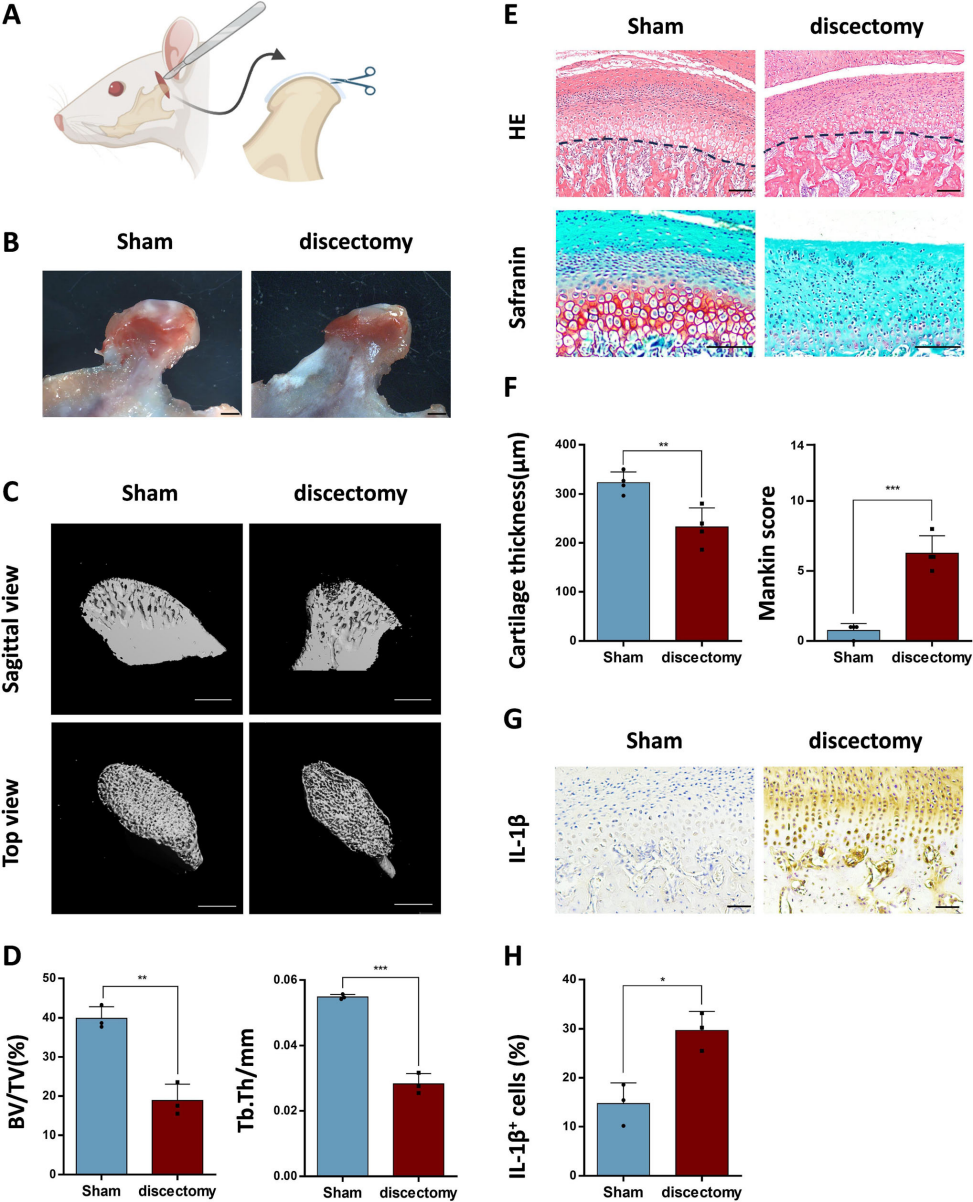
Establishment and verification of rat temporomandibular joint osteoarthritis (TMJOA) model. **A** Schematic illustration of partial discectomy of temporomandibular joint (TMJ) discs. Created in BioRender. B. Condylar tissues of the Sham and the discectomy groups under a stereomicroscope four weeks post-surgery. Scale bar: 1 mm. **C** Rat TMJ condyles collected 4 weeks after surgery were used for Micro-computed tomography (μCT) analysis. Sagittal view and top view. Scale bar: 1 mm. **D** Bone volume fraction (BV/TV) and trabecular thickness (Tb. Th) of the Sham and the discectomy groups. n = 3. **E** Rat TMJ condyles were stained using hematoxylin–eosin (HE) and Safranin O/fast green. The dotted lines in the figure are the dividing lines between the cartilage layers and the subchondral bone tissues. Representative images. Scale bar: 100 μm. **F** Comparison of cartilage thickness and Mankin scores between the Sham and discectomy groups. n = 4. **G** Immunohistochemical staining of Interleukin-1β (IL-1β) in the two groups. Representative images. Scale bar: 100 μm. **H** Quantitative analysis of the percentage of IL-1β positive cells four weeks after surgery in the Sham and discectomy groups. n = 3. Data represented are mean ± SD, *p < 0.05, **p < 0.01, ***p < 0.001, ****p < 0.0001

We created the model of TMJOA by partially removing the articular discs in 6-week-old SD rats (Fig. 1A). Four weeks post-surgery, significant articular disc defects were observed in the condyles of rats in the discectomy group (Fig. 1B). μCT analysis was performed on the condylar tissues. Sagittal graphs of the TMJ condyle subchondral bones indicated an increase in the width of the bone marrow cavity within the discectomy group (Fig. 1C). Compared to the Sham group, both BV/TV and Tb. Th were diminished in the discectomy group (Fig. 1D). Besides, the cartilage layer got thinning, and surface cells were disordered four weeks after discectomy (Fig. 1E, F). Cartilage degeneration was blindly assessed according to the Mankin scores system. The scores in the discectomy group were markedly elevated in comparison to the Sham group (*p* < 0.001, Fig. 1F). Immunohistochemical staining showed that the IL-1β-positive cells in the discectomy group were twice those in the Sham group (*p* = 0.011, Fig. 1G, H). These suggested that we successfully established the model of TMJOA.

### Local injection of PTH restores subchondral bone tissues

**Fig. 2:**
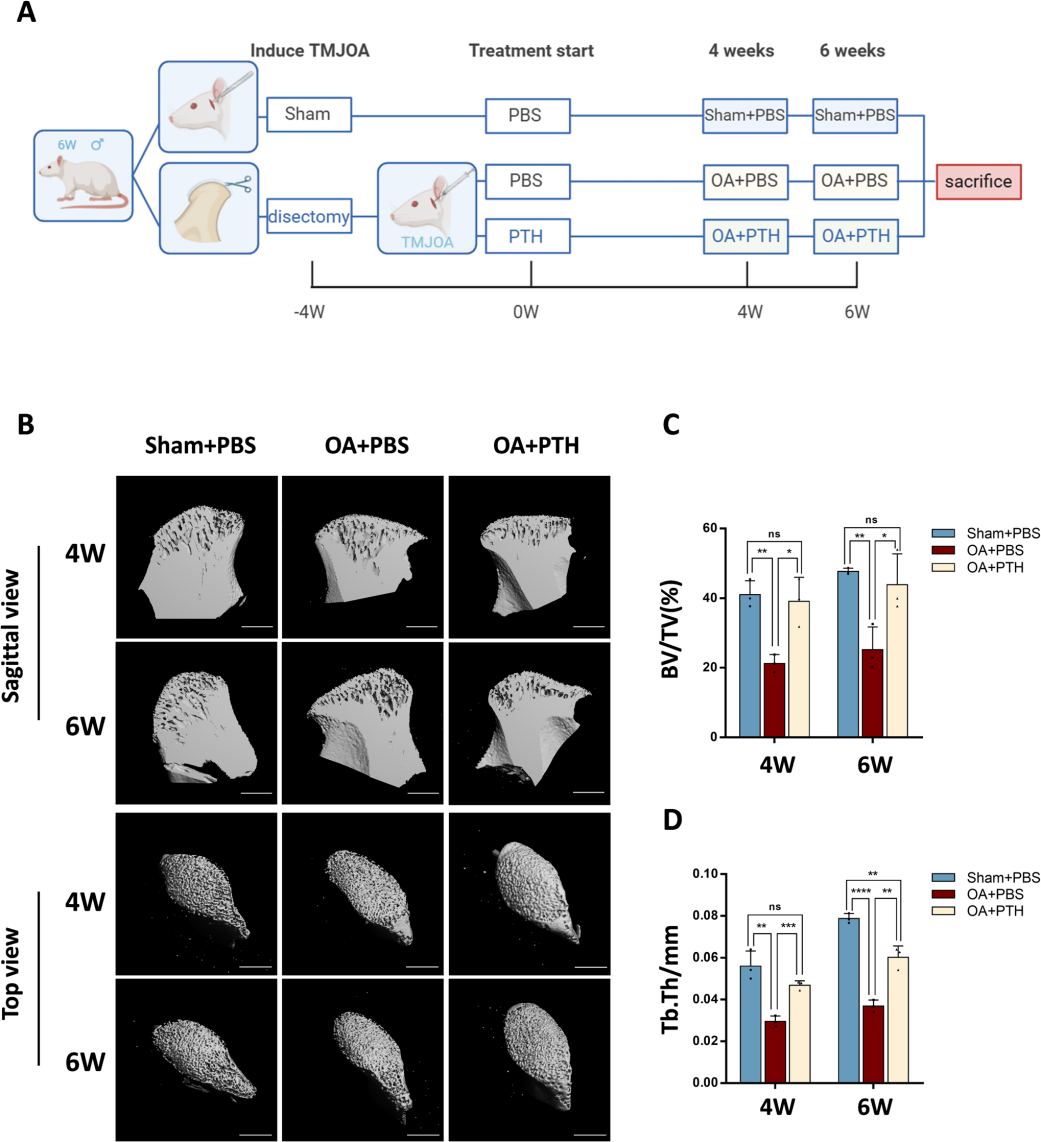
Parathyroid hormone (PTH) repairs the subchondral bones of TMJOA rats. **A** Flow chart of rat experiments. Local injection of PTH in joint cavities began 4 weeks after the operation. Then, condylar tissues were obtained 4 or 6 weeks after PTH treatment. Created in BioRender. **B** TMJ condyles were used for μCT analysis. Sagittal and top views of the condyles. Scale bar: 1 mm. **C**, **D** BV/TV and Tb. Th of the Sham + PBS, OA + PBS, and OA + PTH groups at 4 and 6 weeks. n = 3, data represented are mean ± SD, *p < 0.05, **p < 0.01, ***p < 0.001, ****p < 0.0001, ns, not significant

In our study, treatment began 4 weeks after TMJ surgery once TMJOA had developed. TMJ cavity injections of PTH were administered to the OA + PTH group, while PBS vehicle was given to both the Sham + PBS and OA + PBS groups. These injections were administered for 4 or 6 weeks (Fig. 2A). Bone-related parameters were analyzed to explore the effect of PTH on TMJOA. Images of the sagittal sections of TMJ condyles revealed an augmented bone medullary cavity space in the OA + PBS group relative to the Sham + PBS group. Following 4 or 6 weeks of PTH treatment, the bone medullary cavity volume in the OA + PTH group diminished relative to the OA + PBS group (Fig. 2B). In the OA + PTH group, the condylar BV/TV at 4 and 6 weeks was higher than that in the OA + PBS group. Additionally, the BV/TV in the OA + PTH group was comparable to that in the Sham + PBS group at 4 and 6 weeks (Fig. 2C). The condylar Tb. Th in the OA + PTH group was higher at 4 and 6 weeks compared to the OA + PBS group but was lower than in the Sham + PBS group at 6 weeks (*p* = 0.0053, Fig. 2D). μCT analysis revealed significant differences in two bone parameters between the OA + PBS and OA + PTH groups.

### PTH ameliorates cartilage degeneration and helps restore cartilage tissues in TMJOA rats

**Fig. 3:**
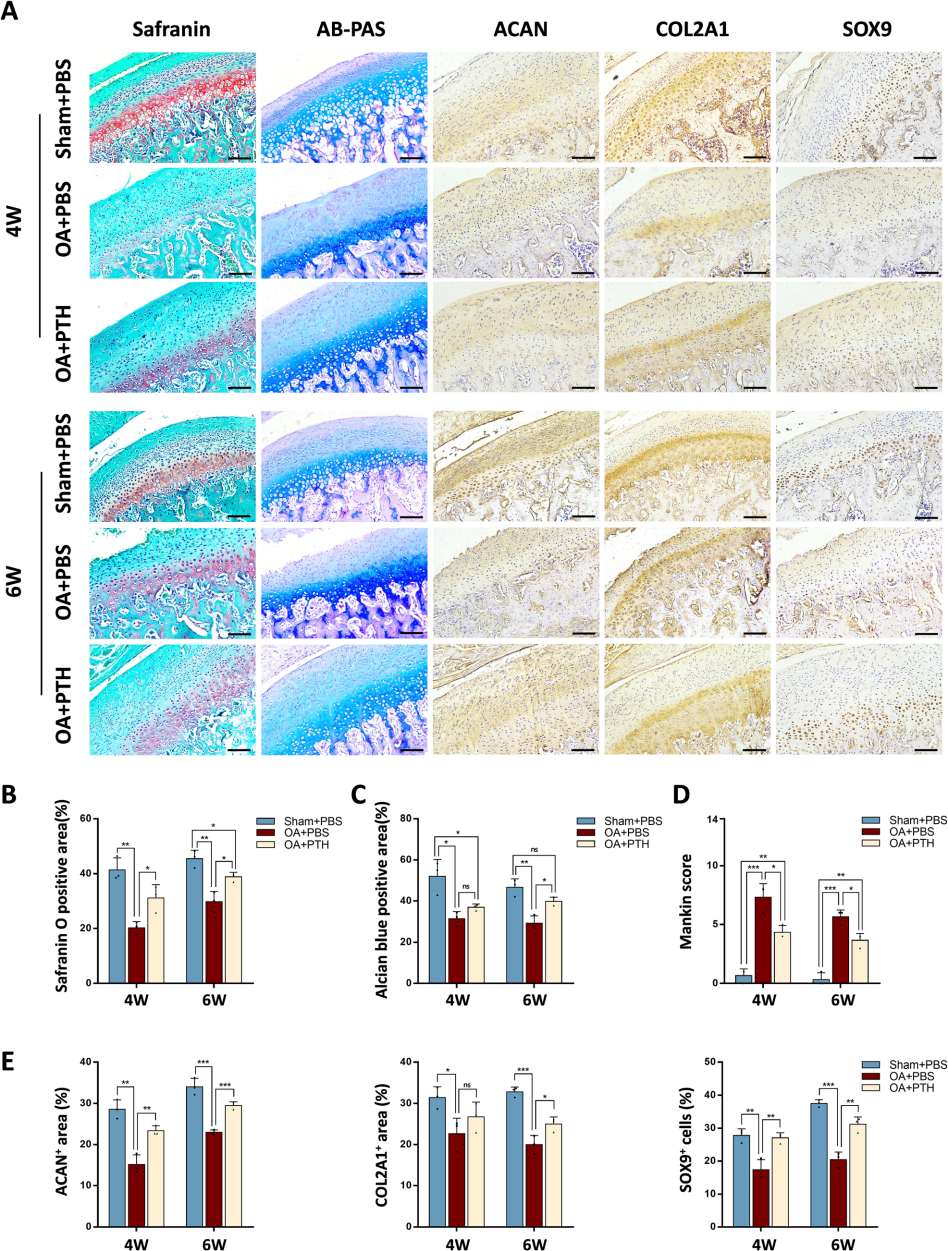
PTH alleviates cartilage degeneration and promotes chondrogenesis in TMJOA rats. **A** The condyle cartilages of the Sham + PBS, OA + PBS, and OA + PTH groups were treated with Safranin O/fast green and AB-PAS staining at 4 and 6 weeks after treatment. Immunohistochemical staining of aggrecan (ACAN), Collagen Type II Alpha 1 (COL2A1), SRY-box transcription factor 9 (SOX9). Representative images. Scale bar: 100 μm. **B**, **C** Quantitative analysis of the proportion of Safranin O and Alcian blue positive areas. **D** Comparison of Mankin scores among three groups 4 and 6 weeks after treatment. **E** Quantitative analysis of the proportions of ACAN and COL2A1 positive areas and percentage of Sox9 positive cells. n = 3. Data represented are mean percentage ± SD, *p < 0.05, **p < 0.01, ***p < 0.001, ns, not significant

We previously demonstrated that PTH could restore subchondral bone tissue in TMJOA rats. Next, we sought to clarify whether PTH injection can restore cartilage tissue in TMJOA rats. Compared to the OA + PBS group, cells in condylar cartilages were arranged more orderly in the OA + PTH group at both 4 and 6 weeks post-treatment. Additionally, the cartilage thickness increased, and the cartilage surface exhibited a marked smoothness in the OA + PTH group (Fig. 3A).

At four weeks post-treatment, the safranin O-positive region was augmented in the OA + PTH group relative to the OA + PBS group (31.12% ± 2.806% VS 20.19% ± 1.345%, *p* = 0.025) (Fig. 3B), and the analysis of condyle Alcian blue staining revealed no significant variations within the two groups (Fig. 3C). Following four weeks of PTH injection in rats, the Mankin scores of TMJ cartilages in the OA + PTH group exceeded those of the Sham + PBS group yet were inferior to those of the OA + PBS group (4.33 ± 0.3333 VS 7.333 ± 0.6667, *p* = 0.0158) (Fig. 3D).

At six weeks post-treatment, both the safranin O-positive area (38.83% ± 0.9465% VS 29.75% ± 2.142%,* p* = 0.018) (Fig. 3B) and Alcian blue-positive area (39.90% ± 1.162% VS 29.18% ± 2.081%, *p* = 0.011) were increased in the OA + PTH group relative to the OA + PBS group. The analysis of condyle Alcian blue staining showed no significant differences between the Sham + PBS and the OA + PTH groups (Fig. 3C). In addition, the Mankin scores of the OA + PTH group were appreciably lower than those of the OA + PBS group (*p* = 0.013) (Fig. 3D).

Immunohistochemical staining showed that at 4 and 6 weeks, the chondrogenic-related genes ACAN and COL2A1 in the OA + PTH group were up-regulated in the matrix. The percentage of SOX9-positive cells was also elevated compared to the OA + PBS group (Fig. 3E). In conclusion, we confirmed that PTH injection could alleviate cartilage damage and promote cartilage formation in TMJOA rats.

### PTH stimulates chondrogenic differentiation of condylar FCSCs *in vitro*

**Fig. 4:**
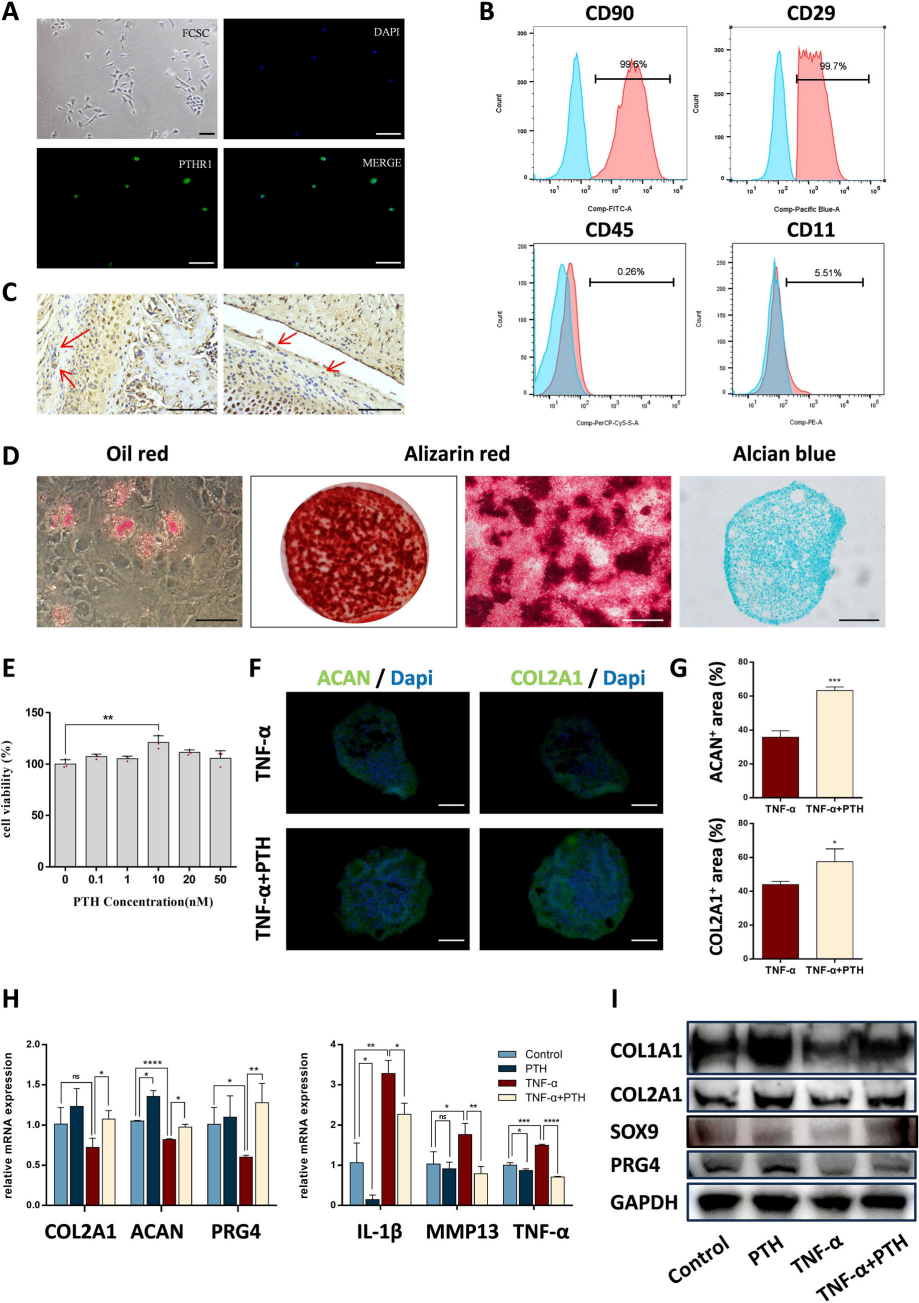
PTH enhanced chondrogenic differentiation of FCSCs. **A** Immunofluorescence staining of PTHR1 in single colonies of condylar FCSCs. Scale bar: 100 μm. **B** Flow cytometry of cell surface markers in condylar FCSCs. **C** Immunohistochemical staining of PTHR1 expression in rat condylar tissues. Scale bar: 100 μm. **D** Multiple lineages of condylar FCSC. Scale bar: 100 μm. **E** The CCK-8 assay was used to assess the cell proliferation under different concentrations of PTH. The absorbance at 450 nm was measured, and the cell viability values were calculated accordingly. n = 3** F** Immunofluorescence staining of ACAN and COL2A1. Representative images. Scale bar: 100 μm. **G** Quantitative analysis of the proportion of ACAN and COL2A1 positive areas. **H** Quantitative polymerase chain reaction (qPCR) was employed to assess the expression of chondrogenic-related and inflammatory markers in condyle FCSCs from the control, PTH, TNF-α, and TNF-α + PTH groups. **I** Western Blot results of condylar FCSCs from the control, PTH, TNF-α, and TNF-α + PTH groups. Data represented are mean ± SD, *p < 0.05, **p < 0.01, ***p < 0.001, ****p < 0.0001, ns, not significant

To investigate whether FCSCs participate in rescuing TMJOA with PTH, we conducted *in vitro* studies. We isolated and extracted FCSCs and analyzed the expression of their surface markers by flow cytometry. Consistent with previous studies [8], the cells expressed CD90 and CD29 while lacking CD45 and CD11 expression (Fig. 4B). PTH-induced cartilage matrix synthesis through the type 1 parathyroid hormone receptor (PTHR1) [21]. We detected the PTHR1 at the surface of FCSC (Fig. 4A), and PTHR1-positive cells were distributed in the condylar surface zone (Fig. 4C). In addition, we confirmed the ability of single clones of FCSCs to differentiate into chondrocytes, osteoblasts, and adipocytes (Fig. 4D).

To further explore the impact of PTH on the chondrogenic differentiation ability of FCSCs under inflammatory conditions, we used recombinant TNF-α protein to simulate the inflammatory environment of the condyle *in vitro* and added PTH intermittently. Previous studies have shown that when PTH was administered at a concentration of 10 nM for the first 6 h of every 48 h *in vitro*, it can promote the proliferation of mesenchymal stem cells [20]. PTH can also increase the migration and adhesion of MSCs [22] and enhance their differentiation into chondrocytes [18]. Based on the homology between FCSCs and mesenchymal stem cells (MSCs), we treated FCSCs with PTH at concentrations ranging from 0.1 to 50 nM for 6 h. Through CCK-8 assays, we observed a significant increase in the viability of FCSCs when treated with PTH at a concentration of 10 nM (Fig. 4E). PTH significantly promoted FCSC chondrogenic pellet formation in the inflammatory environment during induced chondrogenic differentiation. Moreover, the percentages of ACAN-positive and COL2A1-positive areas in FCSC pellets were increased by PTH. (Fig. 4F, G).

After the addition of TNF-α, the mRNA levels of inflammatory factors, including Il-1β, MMP13, and TNF-α, were markedly elevated. The expression of ACAN and PRG4 in FCSCs was diminished. Simultaneously, COL2A1 remained unaffected by TNF-α. In the TNF-α + PTH group, the expressions of IL-1β, MMP13, and TNF-α were dramatically decreased, and the levels of chondrogenic-related markers, including COL2A1, ACAN, and PRG4, were elevated in comparison to the TNF-α group. In addition, the expressions of ACAN were also elevated in the PTH group relative to the Control group (*p* < 0.05) (Fig. 4H). Similarly, in comparison to the TNF-α group, the protein expressions of chondrogenic markers, such as COL1A1, COL2A1, SOX9, and PRG4, were significantly increased in the TNF-α + PTH group (Fig. 4I). Therefore, we revealed that PTH could enhance the chondrogenic ability of FCSCs in an inflammatory environment and alleviate inflammation *in vitro*. PTH could even promote the chondrogenesis of FCSCs in normal conditions to a certain extent.

### PTH rescues TMJOA by promoting the proliferation of FCSCs and chondrogenic differentiation

**Fig. 5:**
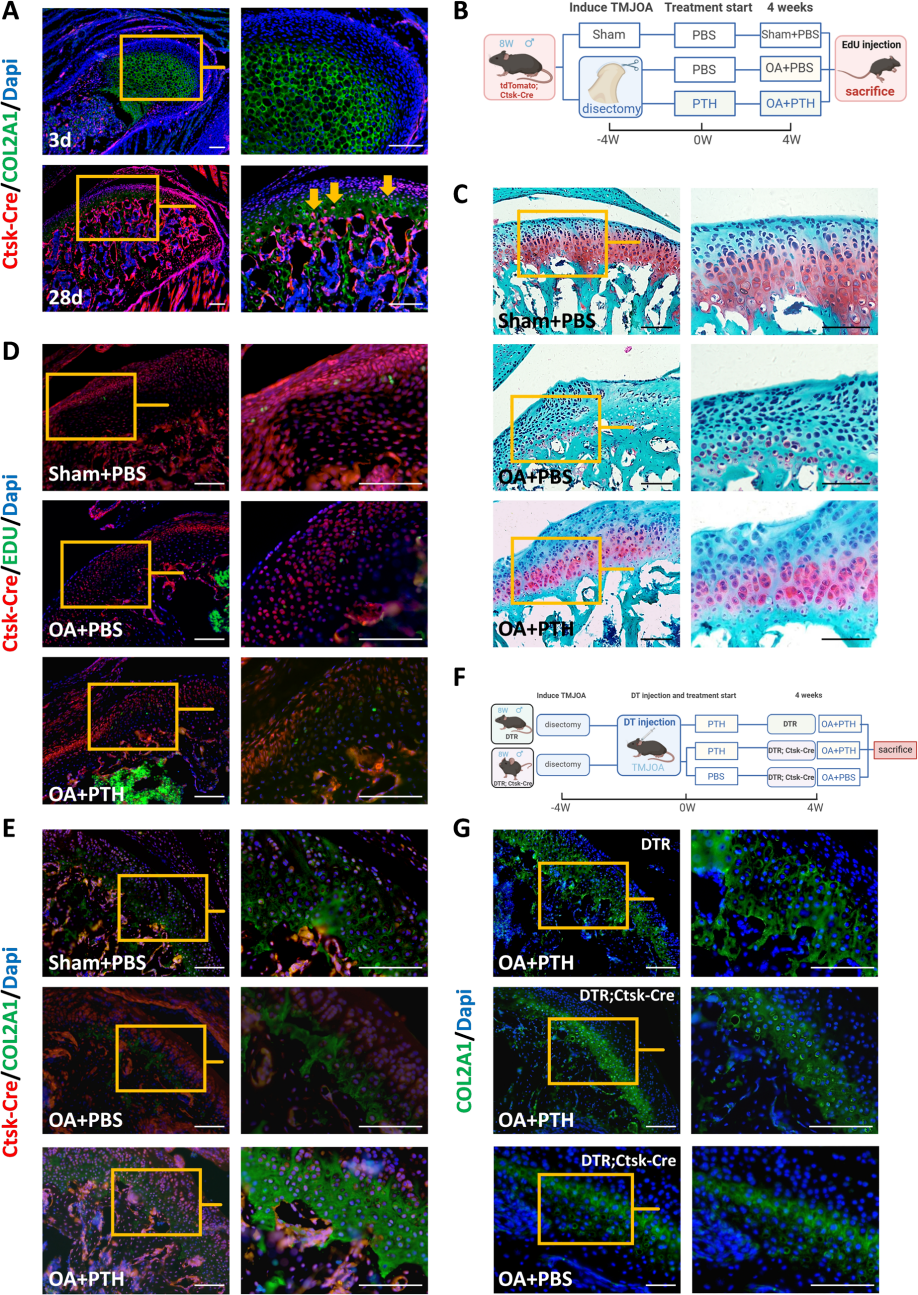
PTH promotes proliferation and chondrogenic differentiation of FCSCs *in vivo*. **A** tdTomato; Cathepsin K (Ctsk)-Cre mice at 3 and 28 postnatal days stained with COL2A1 immunofluorescence. The arrows showed both Ctsk and COL2A1 positive cells in the sublayer of cartilage. Scale bar: 100 μm. **B** Flow chart of tdTomato; Ctsk-Cre mice experiments. Created in BioRender. **C** Safranin O/fast green staining of TMJ condyles in the Sham + PBS, OA + PBS, and OA + PTH groups of the tdTomato; Ctsk-Cre mice. Representative images. Scale bar: 100 μm. **D, E** COL2A1 and EdU immunofluorescence staining in three groups. Representative images. Scale bar: 100 μm. **F** Flow chart of diphtheria toxin receptor (DTR); Ctsk-Cre mice experiments. Administration of diphtheria toxin (DT) selectively eliminated Ctsk-positive cells. Created in BioRender. **G** COL2A1 immunofluorescence staining of TMJ condyles in the DTR and DTR; Ctsk-Cre mice. Representative images

To explore the role of FCSCs in rescuing TMJOA with PTH, we searched for a marker to trace FCSCs *in vivo*. Ctsk can label MSCs in the periosteum of long bones and the mucous membranes of the maxillary sinus floor [23]. We verified that Ctsk can label FCSCs based on the FCSCs belonging to MSCs. In Ctsk-Cre mice, lineage tracing showed that Ctsk-positive cells were exclusively presented in the superficial layer of condylar cartilages 3 days after birth. Then, Ctsk-positive cells were observed in the deep layer of cartilages in 28-day-old mice, and the cells expressed COL2A1 simultaneously (Fig. 5A). This indicated that FCSCs in the superficial zone of cartilages could express Ctsk and gradually migrated to the deep layer, differentiating into chondrocytes expressing COL2A1.

Then, we constructed TMJOA models of tdTomato; Ctsk-Cre mice and treated them with PTH. EdU was intraperitoneally injected 24 h before the mice were sacrificed (Fig. 5B). We found that in the OA + PBS group, the cells in both the superficial and deep layers of cartilage were arranged disorderly. The Safranin O-positive areas had a severe reduction compared to the Sham + PBS group. In the OA + PTH group, the condylar cells exhibited a more organized arrangement, and the cartilage surfaces got smoother in comparison to the OA + PBS group (Fig. 5C). In addition, the condylar cartilage in the OA + PTH group had more Ctsk/COL2A1 and Ctsk/EdU double-positive cells than those in the OA + PBS group. (Fig. 5D, E). The proliferation and chondrogenic differentiation of condylar FCSCs were demonstrated to be enhanced with PTH.

To further confirm the role of FCSCs in PTH treatment of TMJOA, we constructed the DTR; Ctsk-Cre mice. FCSCs can be depleted via administration of DT in condylar cavities (Fig. 5F). After local depletion of FCSCs in the cartilages of the DTR; Ctsk-Cre mice and PTH treatment for 4 weeks, the expression of COL2A1 significantly decreased than that in DTR mice. Moreover, for the DTR; Ctsk-Cre mice, the expression of COL2A1 in the OA + PTH group exceeded that in the OA + PBS group. The efficacy of PTH in rescuing TMJOA was markedly diminished with the partial elimination of FCSCs (Fig. 5G). Consequently, FCSCs were pivotal in the PTH management of TMJOA.

## Discussion

TMJOA was characterized by cartilage degeneration and subchondral bone destruction [24]. Currently, the commonly used TMJOA modeling methods include injection of sodium thiopental (MIA) [25], occlusion disorder [1], aging [26], and partial TMJ discectomy surgery [16, 27]. The injection model differed from human osteoarthritis since MIA is a metabolic toxicant that can lead to chondrocyte death; the occlusion disorder and age model requires an extended duration to manifest TMJOA, and the inflammatory response of occlusion disorder is mild. Discectomy had a good clinical simulation of late symptoms of TMJOA [28]. Thus, we established the TMJOA model by discectomy of discs. In line with a previous study [1], the inflammatory phenotypes were presented 4 weeks after surgery: the superficial layers became uneven, the cartilage thickness decreased, and the safranin O and Alician blue-positive areas were reduced.

Previous studies have shown that PTH alleviated the irregularities in the articular cartilage surface in rats [29–31] and could induce cartilage regeneration in rabbits with full-thickness osteochondral defects [32]. In accordance with prior studies [1], we verified that PTH could attenuate inflammation (decreased Mankin scores) and promote the recovery of cartilages and subchondral bones (thickened cartilages, elevated expression of chondrogenic-related genes, and increased in BV/TV and Tb. Th). Additionally, we found that the expression of certain chondrogenic genes, such as COL2A1, in the condyle tissues did not significantly differ after 4 weeks of PTH injection, but there was an upregulation 6 weeks after PTH treatment. This suggested that extending the duration of PTH therapy might enhance its clinical efficacy.

PTH has been demonstrated to markedly augment the proliferation of bone marrow stromal cells (BMSCs) [22], promoting their differentiation into chondrocytes and facilitating repair after joint cartilage injury [18, 33, 34]. However, no studies have explicitly focused on particular stem cell subpopulations in the superficial layers of cartilage. Our investigation revealed that PTH could enhance the proliferation and chondrogenic differentiation of FCSCs. Our research indicated that PTHR1 was presented on the surface of FCSCs. Previous studies have shown that PTHR1 expression is upregulated in cartilages with progressive osteoarthritis [30], which could benefit us when treating TMJOA with PTH. TNF-α was considered essential in the structural deterioration linked to the advancement of osteoarthritis [35]. We used 10 ng/mL recombinant TNF-α protein to simulate an inflammatory environment *in vitro*, as FCSCs chondrogenesis was significantly blocked at this concentration [19]. However, we found that the expression of COL2A1 was unaffected by TNF-α in FCSCs cultivated in standard media but significantly deregulated during induced chondrogenic culture, which was in line with a previous study [19]. Additionally, chondrogenic-related genes were upregulated, and levels of inflammatory factors were reduced in FCSCs treated with both PTH and TNF-α compared to the TNF-α only group. The ability of FCSCs treated with PTH to undergo chondrogenic differentiation was enhanced during the induced chondrogenic culture. These demonstrated that PTH could decrease the expression of inflammatory factors and promote chondrogenic differentiation of FCSCs under normal or inflammatory conditions.

To track FCSCs *in vivo*, we utilized lineage-tracing reporter mice. Previous studies have indicated that FCSCs can be marked by smooth muscle actin and Glioma-associated homologue-1 [8, 36]. Additionally, Sclaxis can label a specific population of FCSCs, which do not transdifferentiate into bone cells after initially differentiating into chondrocytes. All these markers can only label a heterogeneous population of condylar FCSCs, so we found a new marker of FCSCs in our study. Ctsk could mark BMSCs in the periosteum and cartilage [23]. Since BMSCs and FCSCs are part of the mesenchymal stem cell lineage, we proved that Ctsk could also label condylar FCSCs *in vivo*. For further research, we constructed a model of TMJOA in tdTomato; Ctsk-Cre mice. We demonstrated that in inflammatory environments, PTH can enhance the proliferation and chondrogenic differentiation of condylar FCSCs *in vivo*, potentially offering therapeutic benefits for TMJOA.

We confirmed that PTH promoted cartilage differentiation of FCSCs under inflammatory conditions. However, our study has some limitations and shortcomings. We still lack a detailed understanding of the underlying mechanisms, and further investigation is needed. The PTH signaling pathway is intricate and encompasses multiple interconnected regulatory networks [37]. The PTH-related peptide (PTHrP) / Indian hedgehog (Ihh) pathway is critical in cartilage formation and interacts with the classical Wnt pathway [38]. Inhibition of the classical Wnt pathway has been demonstrated to enhance the differentiation of FCSCs into mature chondrocytes [8], which can reduce condylar cartilage degradation and aberrant subchondral bone remodeling while maintaining or even expanding the FCSC pool [15]. Further investigation is necessary to elucidate the role of the PTHrP/Ihh pathway in the proliferation and differentiation of condylar FCSCs promoted by PTH.

In this work, we used Ctsk to label condylar FCSCs, demonstrating for the first time the effect of PTH *in vivo* on the proliferation and chondrogenesis of condylar FCSCs, restoring the cartilage thickness of TMJ in the inflammatory environment and alleviating TMJOA through the application of PTH. In addition, PTHR1 was presented on the surface of FCSCs, and PTH could also promote chondrogenic differentiation of FCSCs *in vitro*. Currently, the main treatment for TMJOA is to inhibit the progression of inflammation [5], and our study provides a new method crucial for developing and maintaining homeostasis and regenerating condylar cartilage.

## Data Availability

The datasets used and/or analyzed during the current study are available from the corresponding author on reasonable request.
